# Supplementary material for: Ncx3-Induced Mitochondrial Dysfunction in Midbrain Leads to Neuroinflammation in Striatum of A53t-α-Synuclein Transgenic Old Mice
Source: Int J Mol Sci. 2021 Jul 30;22(15):8177. doi: 10.3390/ijms22158177 (PMC8347885; doi:10.3390/ijms22158177)
Supplement: Supplementary file 1 [file ijms-22-08177-s001.zip › ijms-1305584-supplementary.pdf]

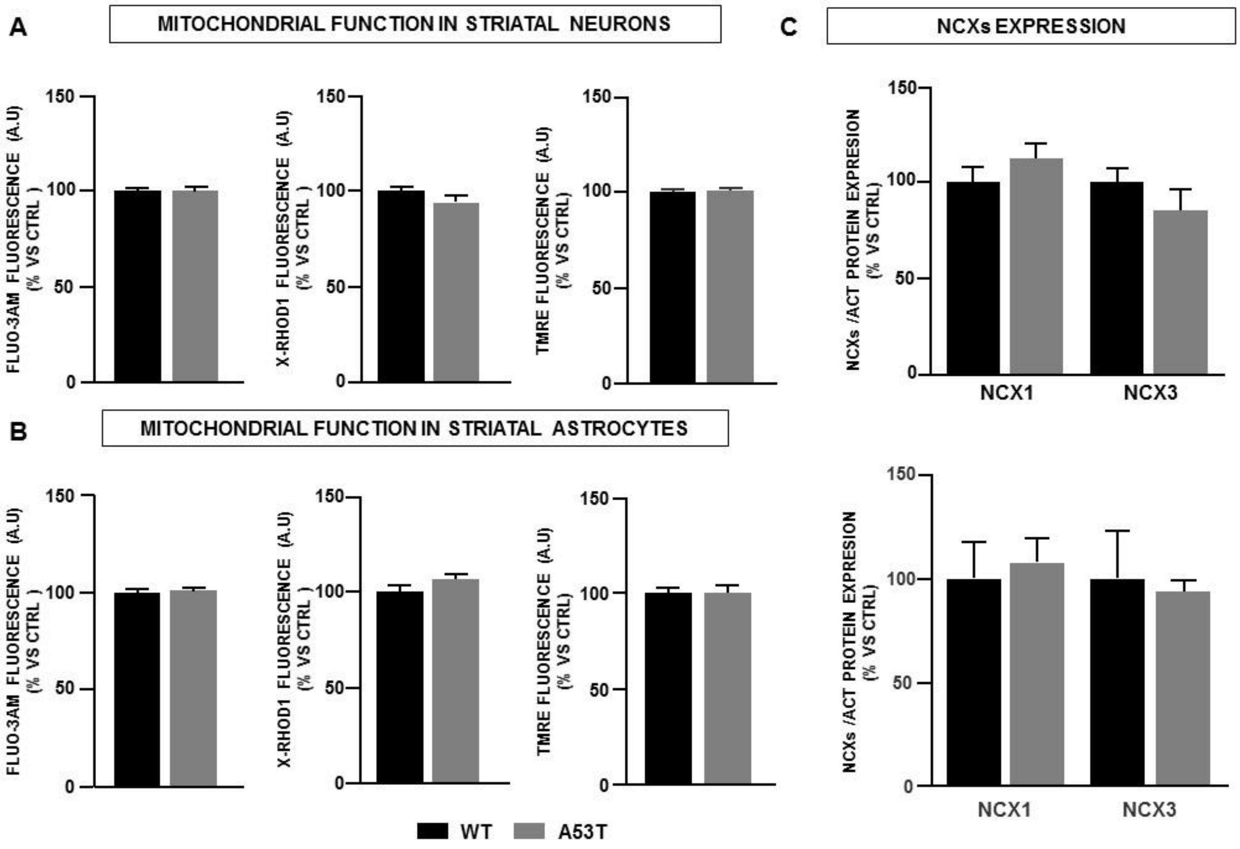

Figure S1: Mitochondrial function and NCXs expression in striatal neuronal cells from A53T α–syn and WT mouse embryos/pups. (A) Quantification of cytosolic calcium concentrations, mitochondrial calcium content and mitochondrial membrane potential, in primary striatal neurons from A53T-α–syn and WT mouse embryos; (B) Quantification of cytosolic calcium concentrations, mitochondrial calcium content and mitochondrial membrane potential, in primary striatal astrocytes from A53T-α–syn and WT mouse pups (P1); (C) Quantification of NCX1 and NCX3 protein expression in striatal neurons (upper panel) and astrocytes (lower panel). In A and B: each bar represents the mean ± S.E.M. of the percentage of fluorescence intensity values of at least 20–30 cells recorded in three independent experimental sessions. In C: each bar represents the mean ± S.E.M of the percentage of the NCX1 and NCX3 protein expression obtained in three independent experimental sessions.
